# Translating a theory-based positive deviance approach into an applied tool: Mitigating barriers among health professionals (HPs) regarding infection prevention and control (IPC) guidelines

**DOI:** 10.1371/journal.pone.0269124

**Published:** 2022-06-03

**Authors:** Ricky Cohen, Anat Gesser-Edelsburg, Arvind Singhal, Shmuel Benenson, Allon E. Moses

**Affiliations:** 1 School of Public Health, University of Haifa, Haifa, Israel; 2 The Cheryl Spencer Department of Nursing, University of Haifa, Haifa, Israel; 3 The Health and Risk Communication Research Center, University of Haifa, Haifa, Israel; 4 Department of Communication, The University of Texas at El Paso, El Paso, Texas, United States of America; 5 School of Business and Social Sciences, Inland University of Applied Sciences, Hamar, Norway; 6 Department of Clinical Microbiology and Infectious Diseases, Hadassah-Hebrew University Medical Center, Ein-Kerem, Jerusalem, Israel; Freelance Consultant, Myanmar, MYANMAR

## Abstract

**Background:**

Although a wide range of intervention programs and methods have been implemented to increase health professionals’ (HPs) adherence with infection prevention and control (IPC) guidelines and decrease the incidence of healthcare associated infections (HAIs), a significant discrepancy remains between the guidelines and their implementation in practice.

**Objectives:**

This study proposes an applied tool based on the integrated theoretical framework of the positive deviance (PD) approach for developing more effective interventions to mitigate this discrepancy.

**Methods:**

A qualitative study guided by the PD approach based on data from two sources: (1) in-depth archival analysis of systematic review articles, and (2) integration and synthesis of findings based on an extensive empirical study we conducted, involving 250 HPs (nurses, physicians, support staff and cleaning staff) from three governmental hospitals in Israel, over 35 months (January 2017 to November 2020).

**Results:**

The barriers faced by HPs were classified into four main categories: (1) individual-motivational, (2) social-cultural, (3) organizational, and (4) work environment and resource-centered. For each barrier, we constructed a set of questions based on the PD approach. For each question, we adapted and applied methodological tools (e.g., in-depth interviews, focus groups, social network maps, video clips and simulations) to help solve the problem.

**Conclusion:**

Translating a theory-based approach into an applied tool that offers step-by-step actions can help researchers and practitioners adopt and implement the approach within intervention programs to mitigate barriers.

## Introduction

Approximately 5% to 15% of hospital patients worldwide contract healthcare-associated infections (HAIs), and the increasing use of antibiotics has led to antimicrobial resistance [1, 2]. HAIs are one of the main causes of death in most countries, with their incidence escalating at an alarming rate [3]. Infection rates in emerging economies are three to twenty times higher than in high-income countries. While hand hygiene (HH) is one of the essential procedures for preventing HAIs, compliance remains a critical problem in many hospitals across the globe [4–6].

In 2002, the Centers for Disease Control and Prevention (CDC) first published its "Guideline for Hand Hygiene in Health-Care Settings" [7]. In 2009, the World Health Organization (WHO) published its “Guidelines on Hand Hygiene in Health Care” as part of a Patient Safety Initiative that addressed aspects of system change, training, education, evaluation and feedback, workplace reminders, and institutional safety climate [8].

Since the release of these official guidelines, national and international governmental and professional organizations have published additional infection control and prevention (IPC) guidelines adapted to various clinical practices, including guidelines for preventing catheter-associated urinary tract infections, intravascular catheter-related infections, surgical site infections, and more [9].

Despite these guidelines and diverse intervention programs, a significant discrepancy remains between the guidelines and their implementation in practice [2, 5, 10, 11]. For over three decades, those researching HAIs have tried to identify HPs barriers in adhering with IPC guidelines and have investigated the effectiveness of targeted intervention programs to increase compliance. Most of these studies have focused on barriers that stem from organizational factors and/or behavioral components [2, 4, 12, 13]. Systematic reviews provide limited evidence of effectiveness, such that most of the barriers faced by HPs in adhering with IPC guidelines have not been addressed [14].

This study relies on the integrated theoretical framework of the Positive Deviance (PD) approach. This approach is used to identify solutions that will enable HPs to overcome the barriers they face in adhering to IPC guidelines.

The PD approach differs from other common problem-solving approaches in that it strives to identify internal solutions derived from the staff within a unit rather than importing external "best practices" [15]. The PD approach identifies the behavioral practices of positively deviant individuals within a community and builds a social network to distribute and implement these practices over time. This approach is based on the premise that in every community there are individuals or groups whose uncommon behaviors and strategies enable them to find better solutions to problems than their peers, while facing greater challenges and having access to the same resources [16, 17].

To date, studies of the PD approach in the field of infection prevention and control have focused mostly on the effectiveness of the approach on HAIs and HH compliance rates [18–23]. A before-after PD intervention study that was implemented in ICUs and non-ICU units across Veterans Affairs (VA) hospitals in the US indicated a reduction in HAIs of 62% (P<0.001) in the rate of Methicillin-resistant Staphylococcus aureus (MRSA) in ICUs, and a reduction of 45% (P<0.001) in non-ICUs [18, 19]. Similarly, a before-after PD intervention study in Billings Clinic in Montana, a healthcare organization of repute in the US patterned after the Mayo and Cleveland Clinic, reduced HAI (MRSA) infections by 84% between 2006 and 2009. The VA hospitals as well as Billings Clinic received high acclaim from CDC analysts for their highly significant statistical declines in their HAI rates on account of the PD intervention [24].

Although PD-centered intervention programs have shown a decrease in infection rates, studies have not investigated how the approach mitigates the barriers between guidelines and their implementation in the field [24–26].

The purpose of this study was to build an applied tool—based on the integrated theoretical framework of positive deviance (PD)—for developing more effective interventions. We provide a step-by-step demonstration of how this framework can address and mitigate HPs’ barriers to adhering with IPC guidelines. This study has two specific aims: (1) to analyze the literature in order to reconceptualize the major barriers faced by HPs to adhere with IPC guidelines, and (2) to propose step-by-step actions using applied methodological tools to mitigate those barriers based on the use of the PD approach.

## Materials and methods

### Study infrastructure

This research is a qualitative study that conforms to the SRQR reporting guidelines (S1 Appendix). The research is guided by the PD approach, based on data from two sources: (1) in-depth archival analysis of systematic review articles, and (2) integration and synthesis of these findings based upon on an extensive mixed-methods study (qualitative and quasi-experimental) we conducted over 35 months (January 2017 to November 2020) that included 250 HPs (nurses, physicians, support staff and cleaning staff) from three governmental hospitals in Israel (Hadassah Medical Center, Rambam Health Care Campus, Bnai Zion Medical Center).

**Fig 1:**
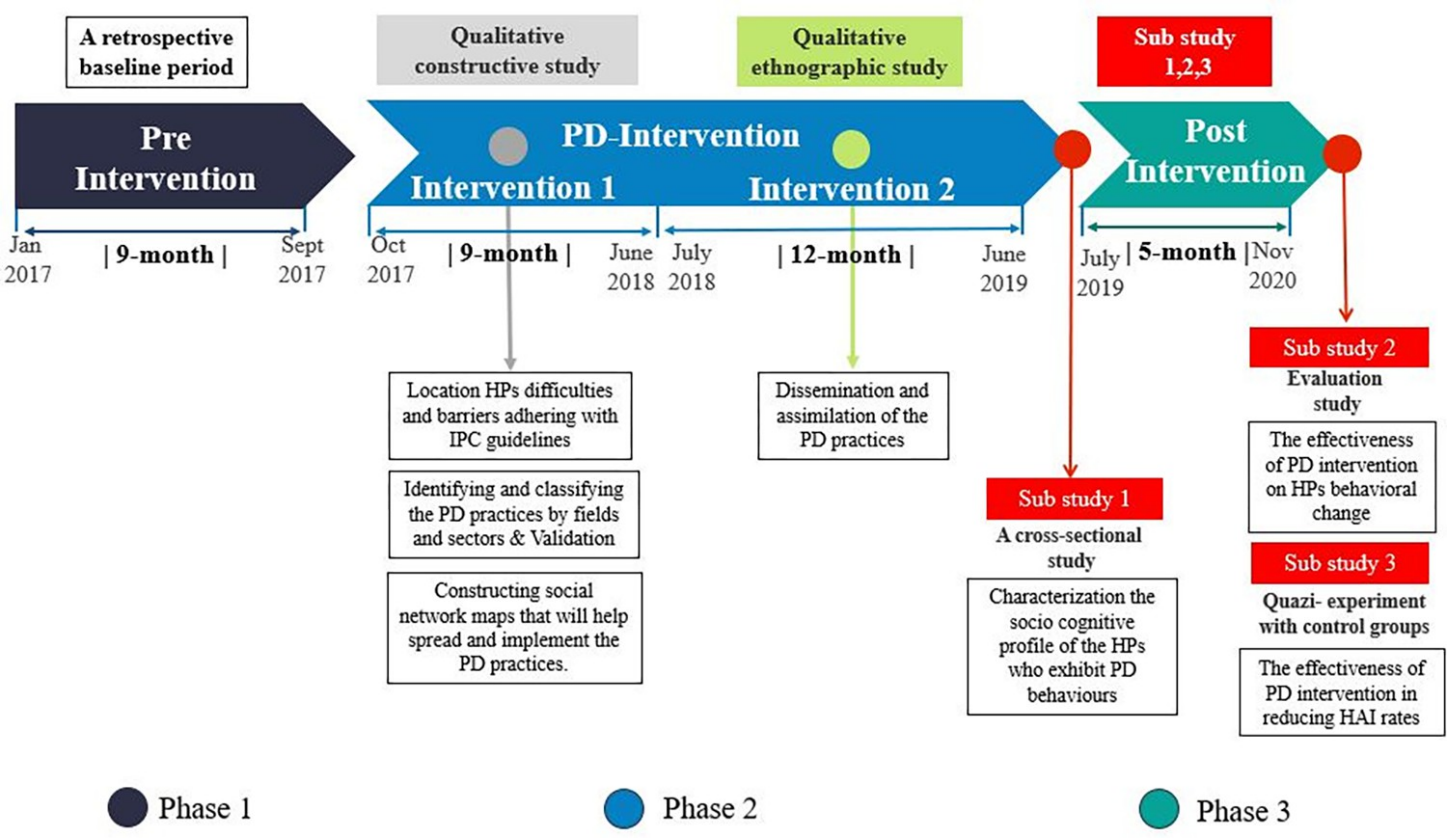
Empirical study design.

**Phase 1:** (Pre-Intervention)–This phase served as a retrospective baseline period only (9 months) for statistical comparisons between pre-intervention incidence rates (IR) and incidence rates during and after intervention (Fig 1).

**Phase 2:** (PD Intervention)–This phase was divided into two periods.

Intervention 1 (9 months) took the form of a qualitative constructive study with the following specific goals: (1) to identify the barriers of HPs at three Israeli governmental hospitals in adhering with IPC guidelines; (2) to identify and classify PD practices for maintaining hygiene according to fields and sectors, and to validate these PD practices in the Infection Control Unit; and (3) to construct applied methodological tools based on the PD approach that can serve as an infrastructure for developing more effective interventions to mitigate HPs’ barriers. We triangulated the data obtained from face-to-face interviews, observations, and videos (Table 1), alternating between conducting interviews and observations and filming videos for periods of several hours during different day shifts. PD behaviors were documented in detail in a field note.

**Table 1:** Research tools used in the empirical study, classified according to hospital intervention departments.

	Bnai Zion Medical Center		Rambam Health Care Campus		Hadassah Medical Center		
Research tools	Internal Medicine B	Orthopedic	Internal Medicine H	Orthopedic	Medical ICU (MICU)	General ICU (GICU)	Total (n)
Interviews	27	24	23	29	35	47	185
Observations and videos	10	9	13	10	15	12	69
Focus groups-based DAD	3	3	2	3	7	5	23
Video-recorded simulations	12	9	9	5	8	10	53

Intervention 2 (12 months)–The specific goal set for this period was to demonstrate how PD practices are implemented and diffused by deconstructing the guidelines for Central Line (CL) insertion at two intensive care units (ICUs) at Hadassah Hospital. This study was conducted using qualitative ethnographic research. CL insertion practices were disseminated by means of simulations demonstrating the order of actions and practices associated with CL insertion. Simulation videos were used for designing and developing activities to disseminate the PD solutions and help community members learn and practice positive behaviors in CL insertion (Table 1) [27–29].

**Phase 3:** (Post Intervention)–Phase 3 was divided into three sub-studies. Sub-study 1 was a cross-sectional study conducted at the beginning of the post-intervention period which aimed to characterize the socio-cognitive profile of HPs who exhibit PD behaviors. Participants first filled out a socio-cognitive characteristics questionnaire, with adjustments made to fit the research topic. After five months they were asked to complete a self-report behavioral change questionnaire (Sub-study 2). This evaluation study sought to examine the effectiveness of the PD intervention in bringing about behavioral change in maintaining IPC guidelines. The questionnaire was built specifically for the study participants based on the PD practices that emerged [30].

Sub-study 2 was conducted at the end of the experimental period, together with Sub-study 3, which sought to examine the effectiveness of PD intervention in reducing the rates of HAIs caused by resistant bacteria and central line-associated bloodstream infections (CLABSI). Sub-study 3 was conducted as a quasi-experimental study with control groups. Incidence rates were calculated based on surveillance data from each participating hospital.

## Research process

### Aim 1: Literature analysis and reconceptualizing barriers faced by HPs to adhere with IPC guidelines

We summarized and integrated the barriers HPs encountered in adhering with IPC guidelines based on four studies: one systematic review, two systematic qualitative literature reviews, and one comprehensive theoretical study [31–34]. Our goal was to discover the diversity of these barriers through a comprehensive examination of the literature over the past two decades. We therefore focused on systematic literature reviews and excluded single studies. Our investigation included all studies published in English from 2011 to 2020 that explored barriers faced by HPs in adhering with IPC guidelines in general (i.e., not specific to a particular practice). We excluded studies conducted specifically in low- and middle-income countries, where the prevalence of HAIs is greater and where most barriers are directly related to a lack of resources due to extreme poverty. The following keywords were used in selecting the studies: "hand hygiene guidelines"; "infection prevention and control (IPC) guidelines"; "health professionals’ barriers"; "healthcare workers’ adherence"; and "healthcare workers’ compliance".

### Aim 2: Step-by-step actions in applying methodological tools to mitigate barriers faced by HPs

To address this aim, we constructed an integrated framework based on the findings of the empirical study conducted in Phase 2, as described above. For each barrier, we systemically applied PD theory to construct open-ended questions. Our purpose was to adapt these questions to a given barrier in order to encourage thinking about applicable solutions. These questions helped us devise the methodological tools for collecting the research data. Through this systematic process, we were able to find applicable solutions for addressing, mitigating, and overcoming barriers.

### Analysis

#### Aim 1

**Fig 2:**
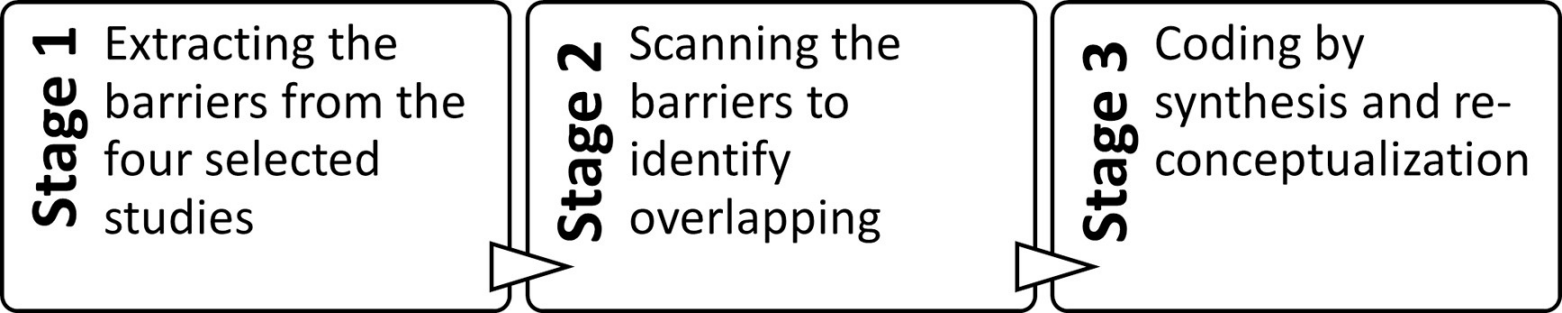
Coding the barriers.

We used the content analysis method to identify the primary and secondary barriers that emerged in the literature. *First*, we extracted the barriers mentioned in each article. *Second*, we scanned all the barriers to determine which overlapped and which were new or defined in a different way. For example, in one article "skill" and "knowledge" were identified as general barriers, while another article simply identified these as "individual" barriers. Accordingly, we classified these two components (skill and knowledge) under the theme of "individual" barriers. *Third*, we coded the barriers using synthesis and reconceptualization, with each main barrier as a primary theme and the secondary barriers as key components described as sub-themes (Fig 2). We added an appropriate description to clarify the specific relationship between the primary and secondary barriers.

Three researchers were involved in coding the barriers based on content analysis of the four articles. Each of them read the analyses of the other researchers and then discussed the analyses until they reached coding consensus (inter-rater reliability).

#### Aim 2

Based on our previous findings from Phase 2 of the empirical study, the *first analysis stage* for Aim 2 entailed gathering data on HPs’ barriers in adhering with IPC guidelines, together with examples of PD practices that addressed those barriers. These data were gathered via interviews, focus groups and observations and subsequently underwent content analysis. In the *second analysis stage*, participants were asked questions about each barrier based on the PD approach. In the *third analysis stage*, their answers were systematically translated to develop methodological tools, which we then implemented during this study to find applicable solutions for each barrier.

### Validity and reliability

The first part of this article (Aim 1) discusses the findings emerging from the integration and synthesis of the barriers to adhere with IPC guidelines—identified from salient systematic review articles published over the past two decades. The second part (Aim 2) is based on a synthesis of the findings of an empirical mixed-methods study that relied on quantitative and qualitative research methods. This synthesis allowed for broad integration between diverse perspectives, leading to a deeper understanding of the phenomenon. To this end, the researchers had to take into account the overall context of the participants’ work, together with the broader framework of the culture in which this work takes place. Qualitative research tools were used to identify and map the implementation and dissemination of PD practices during the intervention phase [35]. We used triangulation (face-to-face interviews, observations, and video) as a strategy to achieve the proposed objectives, increase validity and reliability, and obtain greater scientific rigor. Triangulation makes it possible to mitigate biases resulting from a single analytical perspective [36]. Interviews and focus groups were recorded, transcribed, and logged in a field diary, thus enabling the researchers to examine the reliability of the data and use greater rigor in analyzing the findings.

Moreover, the study participants represented different HP subpopulations (physicians, medical residents, nurses, nurse assistants), thus strengthening the credibility and validity of the findings relative to the studied phenomenon.

We also reinforced the validity of the study by obtaining confirmation from the Infection Control Unit (two physicians and one infection control nurse) regarding the identified PD practices. Throughout the study, the researchers who collected the data via interviews and observations constantly reflected on their evaluations so as to avoid being judgmental or critical and to make sure they were focusing only on discovering PD practices [28].

### Ethics

This study was approved by the ethics committee of The Faculty of Social Welfare and Health Sciences at the University of Haifa (confirmation number 392/17) and by the Bnai Zion Medical Center Helsinki Committee (confirmation number 135–16-BNZ).

All the study participants gave their written consent to participate in the research and to publish its findings. The research does not provide any medical or personal information by which each participant can be identified, thus anonymity is ensured.

## Results

To ensure clarity and explain the temporal sequence of the findings, we divide our discussion of the results into three parts: findings related to Aim 1, findings emerging from identifying, classifying and validating PD practices, and findings related to Aim 2. We also summarize the findings in an integrative table (Table 2) to illustrate the relations between the barriers found in the research and the tools emerging from the PD approach.

**Table 2:** Integrative table showing HPs’ barriers to adhere with IPC guidelines, methodological tools, and examples of applied solutions using the PD approach framework.

Aim 1		PD approach		Aim 2	
Barrier Topic (Main theme)	Key Components (Sub-themes)	PD theoretical conceptualization	PD specific questions	Methodological tools in the PD approach	Applied solution using PD approach
**Individual and motivational**	• Knowledge • Skills • Beliefs • Attitude (Self-protection/Perceived risk) • Memory and attention/forgetfulness • Emotion • Professional role/identity • Skin irritation • Discomfort of personal protective equipment • Complacency • Urgency of patient care • Visible resources /used cues/ reminders	PD is a bottom-up approach.	• Do you follow any unique practice for hygiene not in the official IPC guidelines? • How do you protect yourself, patients, and others from transmitting microorganisms? • Do you have any other ideas for maintaining hygiene? • What motivates behavioral changes in HPs?	• Touring unit’s physical structure of the unit and introduction to shift staff. • Conducting semi-structured personal interviews and focus groups about IPC guideline compliance difficulties, infectious disease risk perceptions, norms, hospital organizational culture. • Asking interviewees to name staff members they believed to be PD.	• Asking PDs why and for how long they had been using the unique practice? • Some claimed they believe the practice helps prevent transmission of infections.
**Social/cultural**	• Peer pressure • Teamwork • Patient expectations • Job and seniority • Norms	PD approach assumes that problems can be overcome using solutions that already exist within communities.	• Do you know any community members with exceptional behavior that promotes hygiene?	• Identifying behavioral practices of PD individuals within the community and building a social network. • Exploring how to ensure that PD practices are feasible using current resources. • HPs more likely to adopt and implement the practices than those from outside.	• HPs completed a behavioral change questionnaire reporting changes in behavior and implementation in their work. • Some HPs added their own tips, thus increasing engagement, accountability and motivation to help to the collective effort.
**Organizational**	• Safety climate • Communication of IPC guidelines • raining programs and auditing • Practical assessment tools • Documentation	PD is a grassroots approach that begins from the bottom up.	• How can organization and managers help community members come together? • think about ideas? • make their voices heard? • give them responsibility to lead the change themselves?	• Pre-study meeting with administration and IPC unit representatives to outline research goals, plan, and PD approach and request cooperation.	• Identifying 23 central line (CL) insertion practices not in official guidelines. • Obtaining IPC Unit confirmation of CL PD practices found. • Disseminating and assimilating PD practices through simulations in which PDs explained thoughts and logic behind their actions. • Hospital decision to implement these practices in official guidelines.
**Work environment & resources**	• Physical environment • Personal protective equipment and infrastructure • Staff shortage • Lack of time	PD is an assets-based approach that identifies and amplifies deviant and variant practices within a community.	• How can existing resources be used to solve HAIs?	• Participants told to seek solutions not in IPC official guidelines and not requiring special resources. • Participants were taught to think of behavioral solutions to overcome barriers inherent in lack of resources.	• Identifying a nurse’s PD practice for preventing contamination when transferring blood and urine specimens.

### Aim 1 findings

Aim 1 sought to identify the primary and secondary barriers faced by HPs. The analysis yielded four main themes: individual and motivational barriers; social/cultural barriers; organizational barriers; and barriers related to work environment and resources. Each of these themes was then broken down into key components or sub-themes.

The following key components were found for the theme of *individual and motivational* barriers: knowledge, skills, beliefs, attitudes, memory, emotions, professional role, skin irritation, discomfort, complacency, urgency of patient care, and visible resources. *Knowledge* reflects the extent to which HPs are familiar with IPC protocols, while *skills* refer to specific skills, such as hand hygiene. The *beliefs* component encompasses what HPs believe about health capabilities, the consequences of implementing IPC guidelines, and the influence of fears and concerns on HP adherence. The *attitude* component covers HPs’ perceptions regarding the value of complying with IPC guidelines in protecting them, their family and patients in all hospital departments. The *memory* component encompasses the times and situations in which HPs are more likely to forget to perform HH (e.g., distractions caused by a heavy workload). The *emotions’* sub-theme covers how HPs feel about hand hygiene and about negative feedback, for instance in the form of sanctions. The component of *professional role and identity* describes the extent to which HPs perceive IPC guidelines as part of their professional role. The *skin irritation and personal discomfort* component refers to the degree of personal discomfort HPs experience when using hand sanitizers and personal protective equipment. *Complacency* may prevent HPs from carrying out good IPC practices regularly. *Urgency of patient care* refers to whether HPs prioritize IPC adherence based upon the degree of urgency in the patient’s condition. Finally, the theme of *visible resources* encompasses the role of memory triggers in IPC adherence, such as placing hand sanitizers at the entrance to hospital rooms.

The theme of *social/cultural* barriers encompasses five sub-themes: peer pressure; teamwork; patient expectations; role and seniority; and norms. *Peer pressure* describes the influence of others, including peers, administrators, patients and relatives, in IPC guideline adherence. *Teamwork* enables some HPs to serve as role models for others, while *patient expectations* can exert another form of pressure. *Seniority and job* can also play a role, in that doctors were identified as the group least likely to engage in hand hygiene. Finally, *norms* refer to the organizational culture, which may be supportive or alternatively may be marked by a lack of openness and a fear of negative consequences.

Several *organizational* barriers were identified: safety climate, communication of IPC guidelines, training programs and auditing, practical assessment tools, and documentation. *Safety climate* refers to the extent to which an organization promotes a climate of safety. Does the management team offer support in following safety guidelines, or are the guidelines ambiguous, changing and impractical? The *communication* component describes how IPC guidelines are communicated. Does the organization offer HPs training and timely feedback regarding IPC guidelines? The *auditing* component refers to whether the organization provides programs and systems to monitor changes in IPC adherence. *Practical assessment tools* include methods such as route control and overcrowding control to reduce contamination risk. Finally, inconsistent *documentation and record-keeping* regarding medical operations and procedures is also a barrier to IPC adherence.

A number of barriers related to HPs’ access to the *workplace environment* and *resources* also emerged from the research. The *physical environment* component included factors such as sink location. Another important barrier was related to *personal protective equipment and infrastructure*, both of which were often of poor quality or lacking entirely. *Shortage of staff members* and *not enough time* were two other components of work environment barriers.

#### Findings regarding PD practices

During the empirical study, which conducted over 35 months (January 2017 to November 2020), a total of 38 HPs were identified as PD individuals, responsible for 70 PD practices that were classified and validated by their respective Infection Control Units and did not exist in the official IPC guidelines. The practices were divided into 16 topics on the care continuum as follows: (1) Removal and replacement of a dressing on a surgical cut; (2) Removal of protective clothing when leaving an isolation room, and performing hand hygiene; (3) Procedure of taking a blood sample; (4) Procedure of sending blood samples to the laboratory; (5) Procedure of central line insertion; (6) Changing dressing attached to the central line; (7) Inserting a urine catheter; (8) Performing suction for a respirated patient; (9) Washing a patient in bed; (10) Sterilizing a stethoscope; (11) Procedure of cleaning the patient’s unit and surroundings; (12) Taking a patient’s urine sample with a urine catheter and sending it to the laboratory; (13) Cleaning the nursing station; (14) Mixing IV meds and carrying them to the patient; (15) Replenishing disposable equipment in a patient’s room; and (16) Instructing patients and families on maintaining hygiene in the hospital [27–30, 37, 38].

The process of identifying, classifying, and validating the positive deviance practices yielded four main findings, corresponding to the four barriers HPs encounter in attempting to comply with IPC guidelines.

In the context of *individual and motivational* barriers, we discovered that PD is a *bottom-up approach* that assumes problems can be overcome using solutions already existing within communities. Every participant answered questions and thus participated in the process of finding and discovering behavioral practices among other community members:

- Do you follow any unique practice to help you maintain hygiene that does not appear in the official IPC guidelines?
- How do you protect yourself, your patients, and others from transmitting microorganisms?
- Do you have any other ideas for achieving hygiene?
- What motivates behavioral changes in HPs?

We learned that successful behavioral change is motivated by the promise of a solution. Moreover, if a community discovers a solution on its own, the members are more motivated to implement it.

In the context of *social and cultural* barriers, we discovered that the PD approach assumes that problems can be overcome using solutions already existing within communities. We determined this by asking participants the following question:

- Do you know any community members with exceptional behavior that promotes achieving hygiene?

By searching for and discovering positive deviant practices, HPs become more involved and aware.

In the context of *organizational* barriers, we discovered that PD is a *grassroots approach* that begins from within rather than being dictated from the top and/or from external experts and additional resources. This finding emerged from asking participants questions such as:

- How can the organization and its managers help community members come together, think about new ideas and make their voices heard?
- How can the organization give community members responsibility for leading the change in their own way?

The organization serves as the gatekeeper for implementing the PD approach and not as the process leader.

Finally, in the context of *work environment and resources* barriers, we discovered that PD is an assets-based approach that identifies and amplifies deviant and variant practices within a community, rather than focusing on what is wrong and fixing it from the outside. Internal change agents provide social proof to their peers that complex problems can be solved without additional resources, as we learned by asking the following question:

- How can solutions be found for HAIs using existing resources only, without additional money and manpower?

### Aim 2 findings

The objective of Aim 2 was to develop methodological tools and to implement them in finding applicable solutions for each barrier. As noted, we used semi-structured interviews and focus groups in which participants were asked to name other staff members who demonstrated positive deviant behaviors for maintaining HH or who proposed ideas for such practices.

For the theme of *individual and motivational* barriers, we asked individuals identified as PDs why and for how long they had been using their unique practice? Some did not think what they were doing was special. It was something they had always done, something they believed was valuable and effective in preventing infections from being transmitted.

***Nurse**: "Every morning I clean the equipment carts with chlorine… not everyone does this but I really think it’s basic because everyone touches it all the time… Two months ago, the Ministry of Health asked that this be included in the guidelines… I did this before, without anyone asking or guiding me…. It also affects our mood when we enter a patient’s room and see that everything is tidy and clean"*.

***Physician:** “Handling medical equipment is a big problem, for example, disinfecting the stethoscope between patients. I always keep disinfectant wipes in my pocket just for this purpose. No one told me to do so. I find it hard to imagine that after examining a patient, I put the stethoscope around my neck without disinfecting it and then move on to the next patient."*

For the theme of *social and cultural* barriers,

As we wanted to deepen the understanding of what distinguishes positive deviance (PD) health professionals from their peers? In the empirical study, respondents were asked to rate (for each practice) the extent to which it changed their behavior and motivated them to implement the practice (in accordance with the following ratings: 1- I did not implement, 2- I implemented partially, 3- I have fully implemented, 4- I have fully implemented and added my own practices). 16.9% of them marked 4 in the ranking which actually means that they are a new group of health professionals who have adopted the positive practices of PDs that were originally identified, and also added additional practices of their own. Therefore, increased their engagement and accountability and encouraged them to be more creative and contribute to the collective effort. To these new group of health professionals, we coined the name and called them "PD boosters" [30].

***Nurse:** "I think it would be good to do this (assimilation of PD practices through simulations) for every action. There’s no question that after this process we developed a more correct and effective procedure for reducing the risk of contamination. It’s really a unique project, I’ve never seen a project like this at a hospital before.”*

***Nurse:** "In retrospect, after you see the final film once or twice, it becomes a habit, you do it automatically and it becomes easier.”*

***Nurse assistant:** “We asked an orderly to use a doll to demonstrate how she washes a patient, with all the knowledge and tips she brings from working in the field… Then other staff members demonstrated it again with her, and you can see they really assimilated her action; it’s amazing.”*

To examine the theme of *organizational* barriers, we met with administration representatives from the Infection Control Unit before we began the study. We outlined the research goals, plan, and PD approach and requested their cooperation. After our research in the intensive care units, we identified 23 central line (CL) insertion practices that were not in the official guidelines. These PD practices were confirmed by the Infection Control Unit and then disseminated and assimilated through simulations. During the practical demonstration, the PDs explained their thoughts and the logic behind their actions. As a result, the hospital management and the Infection Prevention Unit decided to incorporate these practices in the official guidelines [27].

***Physician:** “Seeing what these doctors do in the video and understanding the risk of central line contamination is definitely an assimilation process that probably cannot happen in any other way, and I can tell you that even though we have great relations with the staff, it’s been years since we’ve seen an assimilation process transmitted in this unusual way.”*

***Physician:** “It’s amazing to see how this process mirrors how staff members approach things. At the end you can see the change in how people assimilated, implemented, and talked about what they do.”*

To examine the theme of *work environment and resources* barriers, we asked participants to seek solutions that are not found in the IPC official guidelines and do not require special resources. They were taught to think about behavioral solutions for overcoming the barriers inherent in a lack of resources.

Here is one PD practice identified: Following the accepted procedure, a nurse took blood and urine specimens from a patient and then placed the test tubes on a desk near the door of the patient’s room. Then she removed her gloves and robe, performed HH, and brought a special plastic bag for transporting the specimens. She then placed her hand inside the bag, returned to the room entrance, picked up the specimens, put them in the bag and tied it at the top, without any direct contact between the specimens and her hands or the outside of the bag. Next, she placed the bag holding the specimens in the vacuum tube collection system. This procedure does not contaminate the environment, her hands or the laboratory technician and does not require any special resources [28].

***Nurse:** "When I’m in a patient’s room and I finish taking blood or urine, I leave the specimens on the shelf at the entrance to the room. I then remove my robe and gloves, do hand hygiene and return with the plastic bag in which the tests are sent. I place my hand inside the bag and pick up the samples from the inside, so I do not have any direct contact with the samples"*

Table 2 is an integrative table illustrating the interrelations between the barriers found in the research, the tools emerging from the PD approach and their practical implementation. The barrier topic and its key components appear in the first two columns. The third and fourth columns list the theoretical assumptions and specific questions for the PD approach relative to each barrier topic. The fifth and sixth columns give examples of methodological tools and the applied solutions emerging from the empirical study.

## Discussion

Over the past three decades, a wide range of intervention programs and methods have been implemented to increase adherence of HPs with IPC guidelines and to decrease the incidence of HAIs. As indicated by the findings, the literature describes various types of barriers that must be addressed both theoretically and methodologically in order to mitigate them [31–34]. Moreover, in a recent systematic review Mitchell et al. (2020) sought to describe the strength of IPC guideline recommendations published in the last decade. These researchers point out the problems inherent in the existence of multiple IPC guidelines with high variability in terms of strength of evidence, diverse guideline developers, and diverse grading metrics used to develop recommendations [9].

The purpose of this study was to build an applied tool—based on the integrated theoretical framework of positive deviance (PD)—for developing more effective interventions. We provide a step-by-step demonstration of how this framework can address and mitigate HPs’ barriers to adhering with IPC guidelines. This study has two specific aims: (1) to analyze the literature in order to reconceptualize the major barriers faced by HPs to adhere with IPC guidelines, and (2) to propose step-by-step actions using applied methodological tools to mitigate those barriers based on the use of the PD approach.

As noted, in this study we conducted a mapping of 38 new PD practices on the care continuum that have in common that they are not found in the official IPC guidelines and that they are related to common daily actions such as removal of protective clothing when leaving an isolation room and taking a blood sample.

The adaptation of the PD approach to address barriers to adhere with IPC guidelines, as illustrated in this article, is neither intuitive nor ad hoc. It is theoretically and conceptually focused on solving complex problems related to human behavior. Even when a problem stems from a lack of resources, something that is seemingly unrelated to human behavior, the PD approach informs and encourages community members to find solutions on their own. These solutions represent creative ways of mitigating the barrier or of finding an alternative that bypasses the problem.

**Fig 3:**
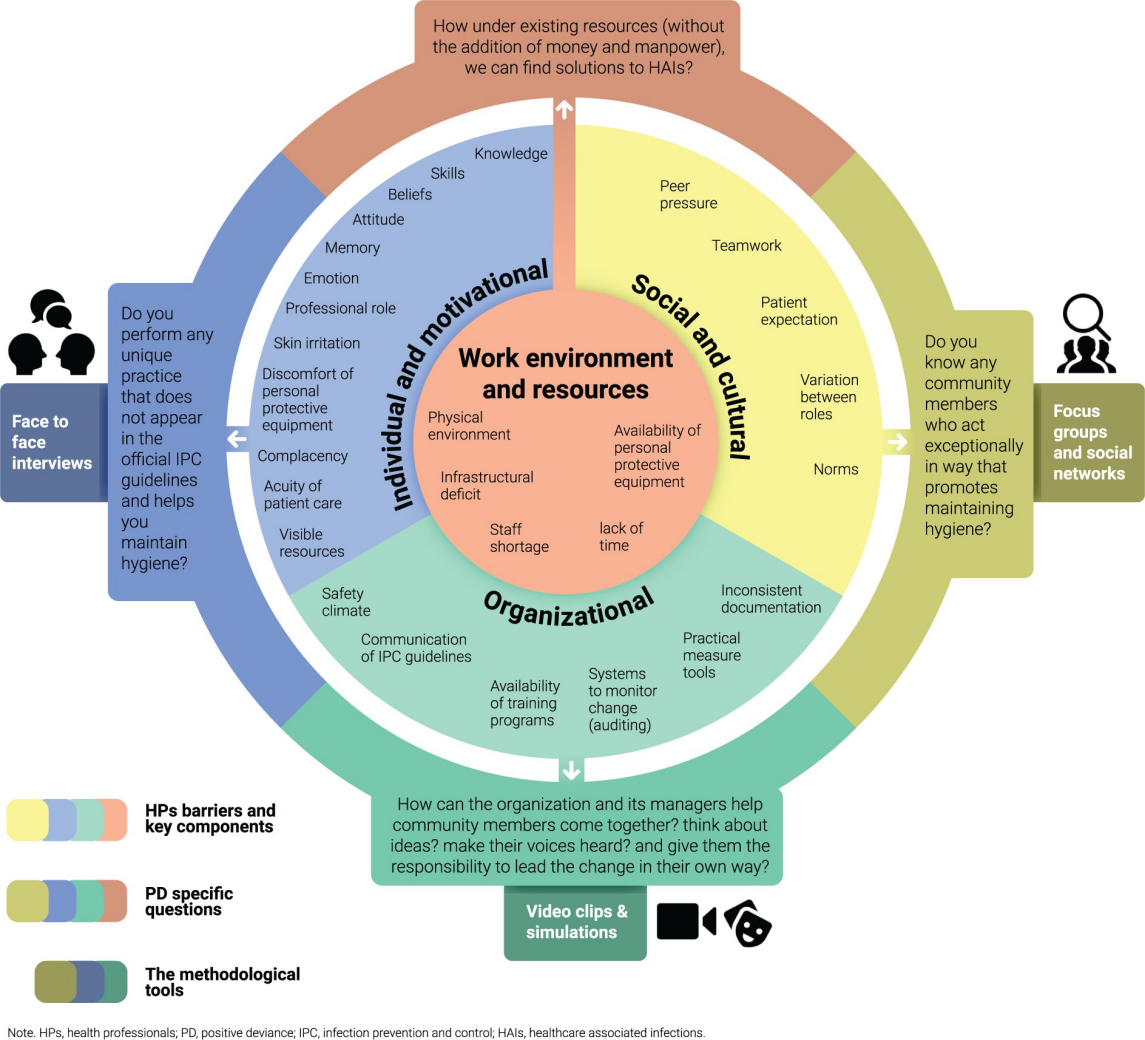
Applied PD tool.

In this article, we described a methodological tool that can be applied systematically from the individual level to the organizational level. As our article demonstrates, the PD approach reverses the accepted order of thinking. It begins by examining barriers on the individual rather than the organizational level and then focusing on what works within the spectrum of available resources. Thus, it lends itself to expansion to the community and organizational levels (Fig 3). The PD approach offers a holistic view of the problem and its complexities, including paying attention to the barriers found in the literature and providing pathways to mitigate them.

To understand how the PD approach deals with the various barriers, we chose to ask questions that can be used as a roadmap and provide an in-depth understanding of the problem. We focused on “who” and “how” questions rather than on “why” or “why not” questions [16].

For barriers identified as individual and motivational in the literature, the PD approach calls on every individual to participate in finding and discovering behavioral practices that may work for others in the community. By being asked to think about community members who they believe have found ways to solve the problem, these people begin engaging in problem-solving from the outset. This activates the existing social network so that everyone on the unit becomes involved in a naturally unfolding process [16].

For barriers identified as socio-cultural, the PD approach calls on community members to make their voices heard while observing and thinking about the unusual yet effective behaviors of other community (or unit) members. Our findings show that not only do community members implement the PD practices they identify among others, they are also encouraged to reflect on and propose additional practices of their own. In other words, the approach encourages more individuals to develop additional PD practices in a virtuous upward spiral [30]. This finding supports the premise on which the PD approach is based: When the solution comes from within the community and not from external experts, the rest of the community tends to adopt the solutions and implement them more strongly. Community members are less likely to oppose change and more likely to accept and apply the wisdom emerging from the system.

For barriers identified as organizational, the PD approach calls on the organization to serve as a gatekeeper for ideas and practices. The organization allows the process to take place without driving the process or drawing upon organizational resources [32, 39, 40]. As the findings show, in our study the hospital management and Infection Prevention Unit decided to integrate and implement the discovered PD practices as part of their official guidelines. Doing so was easy as the solutions were generated internally and came from the bottom up, and the HPs actually practiced them along their care continuum [27].

In terms of barriers identified as work environment and resource-centered, the approach encourages problem-solving using existing resources. The premise is that the solutions to the most complex problems can be found among frontline unit members who are steeped in their work environment and aware of the available resources. Internal change agents serve as social proof to their peers that complex problems can be solved without additional resources. Given that solutions are generated locally and distilled through concrete action steps, they are more likely to be owned and sustained by potential adopters [17, 28].

Our findings indicate that written guidelines cannot be totally comprehensive as they fail to account for the dynamic nature of the work and therefore it is hard to translate them into the work environment, as guidelines cannot address all the "gray areas" on the care continuum. The movement of staff members between tasks is complex and identifying some of the specific situations when hand hygiene needs to be performed is a challenge.

Moreover, it is important to emphasize that the scientific approval for these practices has been given by experts in the Infection Prevention Unit, as we believe should be done by each department and hospital according to their needs [27, 28]. Therefore, the idea is use this approach to create "unwritten guidelines" that are derived from actual people and implemented in the medical unit’s work environment, rather creating never ending new guidelines. Precisely for this reason, the PD approach is very effective because it addresses the specific missing parts on the care continuum that the same community faces. Because these solutions come from the community, it is very likely that people within the system will be more open to adapting them [29, 30].

It should be emphasized that we are not suggesting ignoring existing guidelines, on the contrary, they are the scientific building blocks that need be used in practice. The article’s contribution is a tool kit that can be used to minimize the existing ambiguity between the written guidelines to the work in the field. Community involvement in building the infrastructure will lead to more openness and a multi-systemic effort to reduce infection rates in hospital units.

In light of the ongoing global COVID 19 pandemic, governments and health systems globally and in Israel are facing barriers from the general public with regard to adhering to guidelines, and taking steps to prevent infection from a virus that threatens our lives [37, 41]. This phenomenon is occurring at the macro level, versus HPs confronting HAIs in closed-door settings within hospitals at the micro-level, emphasizing the complexity and importance of seeking additional solutions tailored to contemporary needs, but based on existing community assets.

Gould et al (2022), in their new paper titled "The problem with ‘My Five Moments for Hand Hygiene‴, claim that it is not always possible to implement the Five Moments for all patients all the time. Patients have widely differing needs in diverse settings, and the Five Moments do not adapt well to all these many differences and may overlook barriers that can reduce HH adherence [42]. These findings are also consistent with a rapid qualitative evidence synthesis, recently published by Cochrane (2020) in the light of COVID19, which found that HPs often feel unsure as to how to adhere to local guidelines when they were lengthy and ambiguous, or do not reflect national or international guidelines. They could feel overwhelmed because local guidelines are constantly changing, which leads to increased workloads and fatigue [34]. Since contemporary literature began to internalize that the range of dynamic situations that occur on the care continuum in different settings with different patients, it is clear that one guideline does not fit all. In fact, the questions are designed to pave the way for healthcare professionals to find the answers that are right for them.

Therefore, an approach like the positive deviance which is based on the wisdom of the community and encourages it to find applied and creative solutions tailored to its needs, is a versatile and flexible approach that may maintain positive outcomes for the long run.

### Limitations and future research

One limitation of this research is that we were not able to address all the barriers mentioned in the literature. For example, "skin irritation" that found as one of the key components which belong to individual and motivational barrier topics, or "staff shortage" that found as one of the key components which belong to the work environment and resources. It should not be overlooked that these barriers pose a daily challenge in the field, specifically barriers that directly related to a lack of resources that were excluded from the study, as we did not have to deal with them in our study.

Nevertheless, we were able to incorporate the key barriers reported in systematic literature reviews conducted over the last two decades. Moreover, since the study implemented a PD intervention in a specific hospital department, it is possible that HPs in other departments would have found additional solutions on their own. Furthermore, the PD approach holds that solutions are always found within every community member and may vary from one setting to another.

Another limitation is that this study was completed before the COVID 19 outbreak, so there is no specific reference in this article to barriers that might have developed in this context, although the last qualitative evidence synthesis of the Cochrane pointed to those barriers [34].

The literature offers a wide range of intervention programs intending to raise adherence of HPs with IPC guidelines. Yet there are not enough detailed guidelines and applied methodologies for designing interventions [43] that mitigate these gaps between the guidelines and field implementation. Translating this theory-based approach into an applied tool that provides step-by-step details for action plans can help practitioners and researchers adopt and implement the same intervention programs, thereby mitigating these gaps. Further studies should focus on evaluation studies that examine whether the questions formulated in this model actually lead to finding new practices. In addition it may be that applying the model in different healthcare settings will raise other guiding questions that can be further added to this model.

## Supporting information

S1 Appendix Standards for Reporting Qualitative Research (SRQR). (DOCX)
